# Flying fast improves aerodynamic economy of heavier birds

**DOI:** 10.1038/s41598-024-56325-6

**Published:** 2024-03-27

**Authors:** Charles M. Bishop, Lewis G. Halsey, Graham N. Askew

**Affiliations:** 1 School of Natural Sciences, Bangor University, Bangor, LL57 2UW UK; 2 School of Life and Health Sciences, University of Roehampton, London, SW15 4JD UK; 3 School of Biomedical Sciences, University of Leeds, Leeds, LS2 9JT UK

**Keywords:** Animal migration, Behavioural ecology, Ecophysiology, Evolutionary ecology, Biomechanics

## Abstract

A paradox of avian long-distance migrations is that birds must greatly increase their body mass prior to departure, yet this is presumed to substantially increase their energy cost of flight. However, here we show that when homing pigeons flying in a flock are loaded with ventrally located weight, both their heart rate and estimated energy expenditure rise by a remarkably small amount. The net effect is that costs per unit time increase only slightly and per unit mass they decrease. We suggest that this is because these homing flights are relatively fast, and consequently flight costs associated with increases in body parasite drag dominate over those of weight support, leading to an improvement in mass-specific flight economy. We propose that the relatively small absolute aerodynamic penalty for carrying enlarged fuel stores and flight muscles during fast flight has helped to select for the evolution of long-distance migration.

## Introduction

The rate at which volant animals expend energy during flight is dominated by changes to their mass and airspeed^1^. This is particularly important for long-distant avian migrants that put on large amounts of fuel during pre-migratory hyperphagia and need to arrive at their breeding/wintering grounds at an appropriate time. For example, great snipe *Gallinago media*^2^ have been recorded flying non-stop for 68 h, covering 5863 km from Sweden to equatorial Africa at a speed of 24.3 m s^−1^, with minimal tailwind assistance. Their exact fuel loads are not known precisely, but have been estimated to be a maximum of between 37% and 62.5%^3^, demonstrating a time-reducing high speed flight while heavily loaded. Such migratory behaviour creates a paradox of avian flight because increased body mass is presumed to increase the energy costs of being airborne.

However, our understanding of the interactive effects of speed and weight on flight metabolism and energetics is limited and effectively based on a small number of constrained flight studies in laboratory wind tunnels. These experiments indicated very low increases in flight cost with considerable additional body mass (hypo-allometric scaling of *M*^<1.0^); although no clear explanation was apparent and the researchers could only surmise that this was due to a hypothetical increase in flight muscle mechano-chemical efficiency (red knot *Calidris canutus*, scaling of *M*^0.35^)^4^ or some other speculative physiological adaptation (barn swallow *Hirundo rustica*, scaling of *M*^0.58^)^5^. One field-based study exists and, in complete contrast to the laboratory work, their data showed a very high cost of carrying extra loads (hyper-allometric scaling of *M*^6.8^) for pigeons (*Columba livia*) homing back to their loft^6^. We suspect that this free-flight study most likely suffered from several important confounds, such as drag effects from back-mounted payloads^7^ and weighted pigeons breaking from the flock and returning much later. The literature, then, is missing a robust, repeated-measures experiment on free-ranging birds flying at similar speeds but with different body masses to test whether the additional energy costs of flying when heavier are very small as indicated by laboratory experiments, or are hyper-allometric as might be traditionally interpreted from earlier aerodynamic publications (scaling of *M*^1.25^)^1^. We address this by undertaking a novel design of loading experiment on free-flying homing pigeons which utilised thin ventrally located weight and a low profile and light-weight ECG data recorder; we herein report our findings and provide a new insight into how birds cope with additional weight.

## Results

### Response of heart rate to additional weight

**Figure 1:**
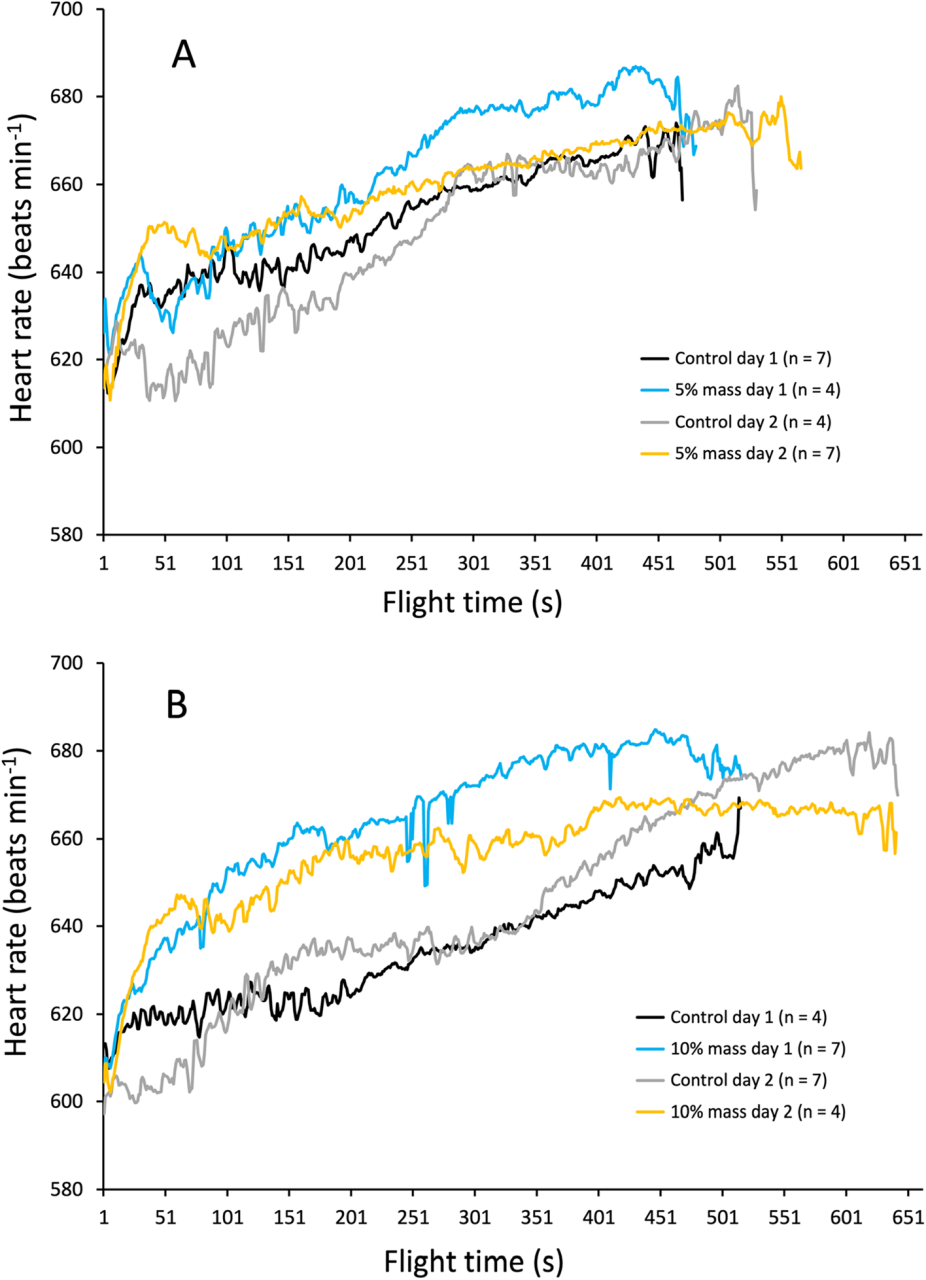
Mean heart rate of 11 pigeons released 10.4 km from the home loft as a flock once a day and plotted against median flock return time. The design is within-subjects—the birds acted as their own controls over 2 days. (**A**) Four experimental birds with 5% added mass on day 1, and 7 experimental birds the next day. (**B**) Seven experimental birds with 10% added mass on day 1, and 4 experimental birds the next day.

**Figure 2:**
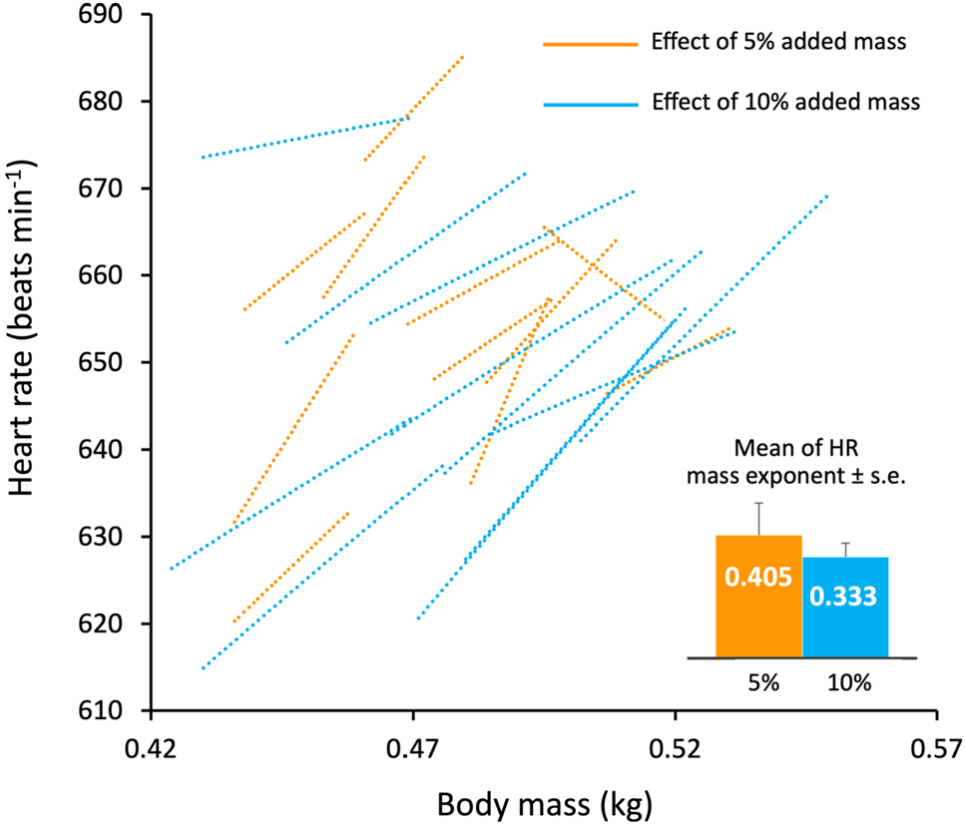
Mean heart rates of 11 flying pigeons against flight body mass, with lines drawn between the control and weighted flight for each bird, where weighted flights involved carrying either an additional 5% body mass (orange) or 10% (blue).

The pigeons were released in a flock 10.4 km from their loft and flight energy expenditure was assessed by measuring heart rate^8^, using custom made miniature ECG data loggers. Birds were flown twice over two consecutive days in a within-subjects design and data were analysed using a paired Student’s t-test. Half of the flock had no extra load (controls), and the other half a thin lead chest plate of 5% body mass (Fig. 1A). Five days later, this design was repeated but with 10% added mass (Fig. 1B). There were 11 paired flights with working ECG recorders for each weighted treatment. The sensitivity of the heart rate response to the increased weight is clearly visible early in the flights (Fig. 1), although the overall increases above the control values are relatively small. There was a tendency for the heart rate to rise throughout the flight, which may be explained by several possibilities: (a) the time required to reach a physiological steady state following take-off and subsequent climbing flight (b) pigeons may tend to fly faster as they approach their home loft and (c) related to this, during these latter stages, the birds could be flying closer to their critical aerobic speed, thus stimulating a slight rise in blood lactate and/or body temperature. The mean whole flight heart rates of the control pigeons for the 5 and 10% experiments were 648.9 ± 4.58 and 640.0 ± 5.22 beats min^−1^, respectively, with the added loads causing only a small increase in within-subject mean heart rate compared to the control flights: 11.5 beats min^−1^ (t = − 4.331, P = 0.0015) and 21.0 beats min^−1^ (t = − 8.059, P = 0.00001), representing just 1.8 and 3.3% increases for carrying an additional 5 and 10% load, respectively; Fig. 2).

### Calibration of heart rate and estimation of energy expenditure

**Figure 3:**
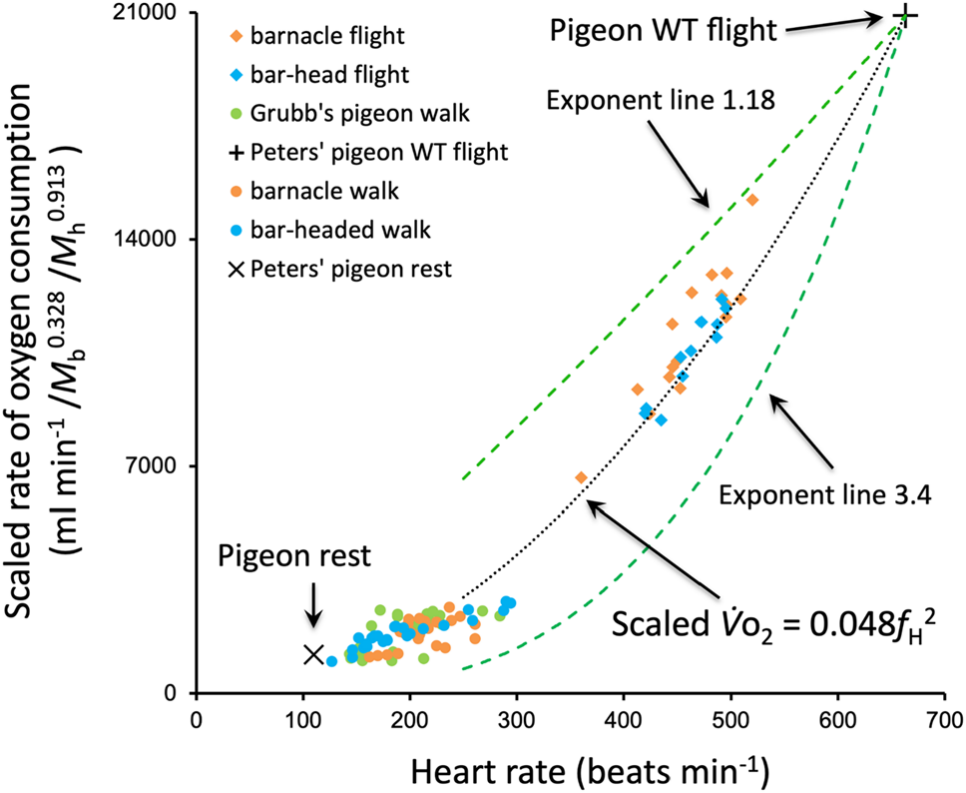
Rate of energy expenditure as rate of oxygen consumption (${\dot{V}}_{{{\text{O}}}_{2}},$ ml min^−1^) adjusted for both body mass and heart mass (as per Bishop and Spivey)^8^. Data plotted were taken from a variety of sources and represent walking and flying barnacle and bar-headed geese^16^, walking pigeons^25^, and resting and flying pigeons^26^. Lines are generated by taking the flying data point for pigeons^26^ and modelling back toward the origin by applying exponential slopes of *f*_H_^3.4^ (lower green), *f*_H_^1.18^ (upper green) or *f*_H_^2.0^ (black). An exponent of 3.4 is required for heart rate changes to match aerodynamic predictions for body mass scaling of power at *U*_mp_ (but see text), while an exponent of 1.18 provides an equidistant shift in the opposite direction from the value of 2.0 (predicted by Bishop and Spivey)^8^ and is also very similar to that measured from the geese while flying and running at different speeds^16^.

Bishop and Spivey^8^ resolved that the mean relationship between rate of energy expenditure (metabolic power; measured as rate of oxygen consumption; $\dot{V}{\text{o}}_{{2}}$ ) and heart rate during primary modes of locomotion is proportional to heart rate squared, especially when differences in body mass and heart mass are considered (scaled $\dot{V}{\text{o}}_{{2}}$ ∝ *f*_h_^2^). This relationship provides realistic values when compared to energetic costs measured in wild free-ranging great frigatebirds *Fregata minor*^9^ and lesser black-backed gulls *Larus fuscus*^10^. Using it to estimate the energy consumption of the homing pigeons (Fig. 3), the average flight costs were increased by only 3.7% and 6.7% (proportional to *M*^0.81±0.21^ and *M*^0.67±0.09^) for 5% and 10% payloads, respectively (n = 11).

## Discussion

Aerodynamic theory predicts that the general relationship between speed and rate of mechanical energy expenditure required to fly is U-shaped^11^ and that, when flying at their minimum power speed (*U*_mp_), i.e. at the base of the ‘U’, a heavier species of bird will have a disproportionately higher aerodynamic power requirement^1,11^. Thus, the minimum mechanical power for a heavier species to fly is predicted to scale with a body mass exponent of 1.17 (power ∝ *M*^1.17^). For an individual bird that increases its payload, the intra-individual relationship is even steeper (as the wings remain the same size), scaling of *M*^1.25^ when the lighter bird (or plane) flies at the *U*_mp_ of the heavier individual^1,12^. As a result, transporting mass through the air at *U*_mp_ should be less economical for heavier individuals. However, our data for the estimate of metabolic power clearly indicate a hypo-allometric scaling relationship and that pigeons can carry extra mass very economically during homing flights. To match the aerodynamic expectation at *U*_mp_ of *M*^1.25^ would require a calibration relationship of scaled $\dot{V}{\text{o}}_{{2}}$ ∝ *f*_h_^3.4^, which in no way fits with the calibration data (Fig. 3).

**Figure 4:**
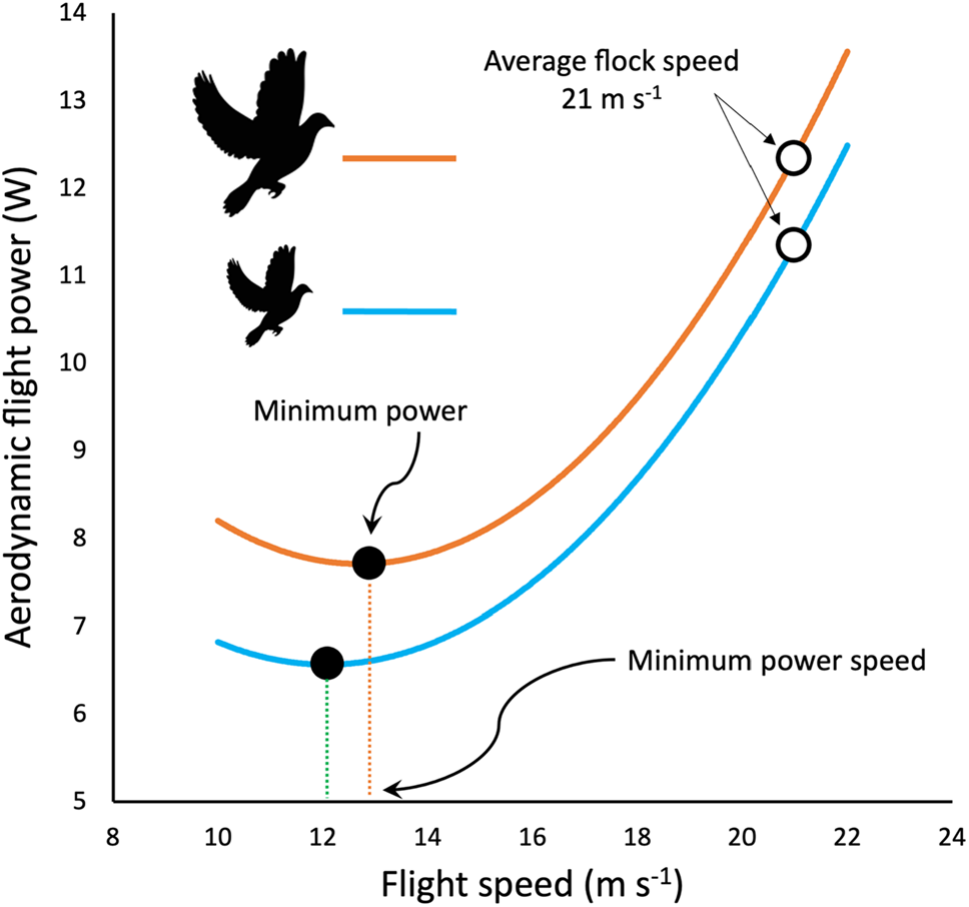
Calculated flight power-speed curves for Homing pigeons with body masses of 461 g (green curve) and 508 g (orange curve), based on the aerodynamic model of Heerenbrink^15^. At minimum power speed (*U*_mp_) for each bird (closed black circles), the relationship between power and mass is hyper-allometric. However, as flight speed increases, the power-speed curves converge and the exponent of power-mass relationship decreases, ultimately becoming statistically significantly hypo-allometric at their typical flight speed of 21 m s^−1^ (open black circles).

While our free-flight results are broadly compatible with previous wind tunnel studies, can these hypo-allometric relationships be reconciled with aerodynamic theory and estimates of mechanical power, without recourse to hypothetical physiological changes? As indicated above, the predictions for the exponent values for the scaling of mechanical power with body mass^1,11^ are calculated on the explicit assumption that birds are flying near the aerodynamic *U*_mp_ of their U-shaped power-speed curve (Fig. 4). However, birds may often fly faster than *U*_mp_, which serves to reduce their journey times and/or increase their flight range, by flying closer to their maximum range speed, i.e. the speed at which the cost of transport is minimal^13^. The average air speed for pigeon flocks flying to the Treborth Loft in subsequent experiments^14^ was around 21 m s^−1^, substantially above the *U*_mp_ of around 12 m s^−1^ calculated by the aerodynamic model of Heerenbrink et al*.*^15^. Applying the Heerenbrink model to pigeons flying at 21 m s^−1^, we obtain a prediction for the scaling of aerodynamic power with body mass proportional to *M*^0.88^, which is similar to the experimental results for our pigeon homing flights of metabolic power $\propto$ *M*^0.67–0.81^.

The use in the present study of thin front-located lead weights and extremely light and low-profile ECG recorders reduced the additional parasite drag effects to a minimum. The rectangular weights wrapped around the upper chest area and did not increase the maximum frontal area of the body, so that only the induced power associated with weight support needed to be increased. Loading in an anterior ventral position could increase role stability around the x-axis but, equally, it might create instability around the y-axis and, in either case, natural abdominal loading is also ventral and arguments for any effect on stability would be similar, whether done artificially or naturally. At higher flight speeds, the relative effect of the added mass becomes diluted relative to the effect of the rapidly increasing parasite drag (proportional to speed squared) and associated additional power (proportional to speed cubed). Thus, at higher speeds, the increase in mass results in a proportionally small additional energy cost as the power-speed curves for the different payloads do not run parallel but converge (Fig. 4). As birds must flap their wings to increase the momentum imparted to the air in order to support the additional weight, there may be associated increases in wing profile power, which could complicate the aerodynamic prediction. However, the present experimental results for the estimated rate of metabolic energy consumption, based on indirectly calibrated measurements of heart rate^16–18^, are consistent with the convergence of the aerodynamic power-speed curves for individual birds with increased payloads flying at speeds higher than *U*_mp_. Foraging and migrating at higher cruising speeds may also enable birds with an increased payload to maintain a high propulsive efficiency, by keeping an optimal Strouhal number (*St* = f A/U)^19,20^, because any increase in wingbeat frequency (*f*) and amplitude (*A*), needed to increase the momentum imparted to the air, will be offset by the increase in speed.

As the pigeons in the present study largely remained as a flock and landed within + 4.5% and + 0.0% of the median time for 5% and 10% loaded pigeons, respectively, our results are controlled against significant variation in flight speed. The power-speed curve in Fig. 4 indicates that the maximum range speed (*U*_mr_) would be slightly higher for the loaded birds, but they either fly at the same speed under 10% load or very slightly slower under 5% loading and generally supports observations of the flock conforming to the Goldilocks principle or speed consensus^21^. Thus, our analysis shows that the extreme hyper-allometric result from the aforementioned previous experiment on homing pigeons^6^ is by no means compatible with the aerodynamic prediction of *M*^0.88^ based on the effects of mass alone. Their published data indicated an impossibly high scaling of *M*^6.8^, which indicates the presence of confounding variables affecting the experimental outcome, such as difference in flight speeds between groups and potential direct and indirect effects of tag parasite drag and harness design. The hypo-allometric results from wind tunnel experiments had the experimental advantage that all the individuals of each species were flown at the same single flight speed, albeit while constraining the birds’ ability to determine their own flight behaviour. Our experimental design also leads to a perspective that directly focusses on body mass effects, but additionally highlights the aerodynamics of high flight speeds when birds are allowed to make their own choice^13^. The air speeds reported for the wind tunnel experiments were 15.1 m s^−1^ and 10.3 m s^−1^, respectively^4,5^, in contrast to their predicted^15^ aerodynamic *U*_mp_ of 8–9 m s^−1^ and 5–6 m s^−1^. Applying the Heerenbrink model, we obtain predictions for body mass exponents for the red knot and barn swallow of *M*^0.73^ and *M*^0.55^, respectively, so the aerodynamic predictions provide hypo-allometric relationships at these relatively high flight speeds. Thus, we suggest that the wind tunnel results, like our own, are best explained by an argument rooted in aerodynamic theory^15^ and do not require recourse to hypothetical physiological changes^4,5^.

Generalising to avian migrants, many species go through pre-migration hyperphagia and begin their journeys with enlarged fuel stores and flight muscles^22,23^. The example of the great snipe^2,3^ may be fairly typical of many long-distance migrants with respect to their degree of weight increase, so we suggest that the present experiment is compatible with these observations of wild migrants and the hypothesis of promoting the evolution of fast migratory flights. A potential confound when comparing the present experimental results with the natural fattening of wild migrants is the effect of lipid deposits on the shape and cross-section of the body and, therefore, the parasite drag. However, while fat stores may be subcutaneous and distributed around the body, the majority of lipid is preferentially located in the abdominal area and behind the flight muscles. Potential effects on pressure and friction drag due to abdominal loading are hard to quantify without further experimentation, however, it is worth noting that the wind tunnel experiment on red knots^4^ used natural fattening of their birds, with one individual increasing their mass by 40%, while flying for many hours with a low mass exponent. It is likely, therefore, that low to moderate fattening will have little effect on maximum cross-sectional or frontal area and body pressure drag, while the effects of higher fuel loads may gradually become detrimental at some point. An extreme example of fattening prior to migration is the bar-tailed Godwit (*Limosa lapponica baueri*) prior to its trans-Pacific flight, during which it approximately doubles its body mass, while both the sizes of the breast muscles and heart increase approximately in proportion (R^2^ = 0.77 and 0.66, respectively)^24^. Thus, while such cardiac hypertrophy is consistent with enhancing sustained flight capability it might be insufficient to maintain maximum airspeeds with this extreme degree of loading. The Godwits did fly faster when heaviest towards the start of their journeys, but this was influenced by selection for favourable tailwinds. Nevertheless, average travel speeds for two birds flying for 6.5 and 8.1 days were an impressive 17.1 and 16.7 m s^−1^, respectively, despite the additional influence of the satellite tags. Thus, along with reduced journey times and the potential to maximise flight ranges^13^, we propose that the relatively low aerodynamic penalty for carrying hypertrophied tissues when flying fast has helped to select for the evolution of long-distance migration.

## Methods

All care and use of the homing pigeons, including homing flights and specific experimental protocols reported in this study were reviewed and approval by the Animal Ethics and Welfare Committee of Bangor University, UK. Further, all methods and procedures were conducted under a UK Home Office ASPeL Project and Personal licence issued to CMB and all experimental flights were performed in accordance with the specific guidelines and regulations contained within the Home Office PPL. A colony of between 50 to 70 homing pigeons (*Columba livia*) were housed in a loft at the Bangor University owned Treborth Botanic Gardens, Bangor, near the Menai Straits (53° 12′ 59.40″ N and 4° 10′ 20.64″ W). Husbandry was carried out daily by qualified animal technicians, with birds provided with water ad libitum but only fed once per day, with an average food weight of 25 g per bird. On the days when birds were released outside the loft, food was made available when the birds returned to the loft. Birds were obtained as chicks of around 3–4 weeks of age from professional breeders and then raised in the loft. The pigeons in the flock ranged from 1 to 3 years of age, with body masses of between 360 and 520 g.

**Figure 5:**
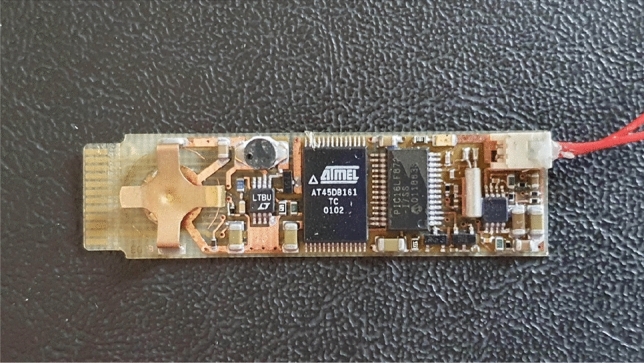
ECG data logger—56 × 15 × 5 mm.

16 pigeons were fitted with lightweight elastic harnesses and gold-plated safety pins (which acted as subcutaneous electrodes) were placed in the skin of the lower neck and rump. This was done at least 1 week prior to experimental measurements, and both could be kept in place for 1–2 months without any long-term negative effects. For the experimental flight days, the 16 pigeons were equipped with custom made miniature ECG recorders (Fig. 5, 56 mm × 15 mm × 5 mm), with a combined harness and logger mass of 5 g (0.96–1.4% of body mass). Electrode wires were connected to the subcutaneous safety pins via miniature 1 mm gold-plated push–pull connectors.

**Figure 6:**
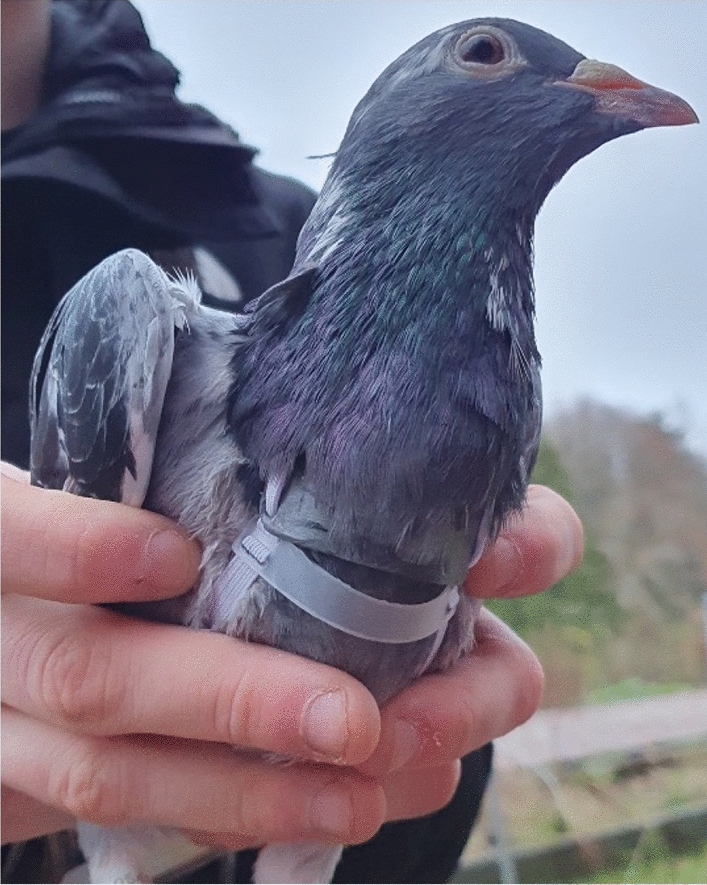
Lead chest weight.

The experimental flights were conducted as pairs with a within-subject design, with all 16 instrumented birds released simultaneously as a flock over a 10.4 km route, including 30 other non-instrumented companion pigeons. All birds were corralled into carriage boxes in the early afternoon before being weighed, instrumented with the ECG loggers, returned to the transport crates and driven to the release site (A55 Malltraeth Flats roundabout—53° 14′ 22.02 N and 4° 19′ 24.47″ W). The release crates were taken out of the car and placed on a wall on the edge of the Menai Strait. Birds were left for 5 min in these open mesh roofed transport boxes before the simultaneous group release. Each bird was flown twice over two consecutive days (13 and 14/02/2003), with half of the instrumented birds on a particular day carrying a thin plastic strip across the chest (62 × 23 × 1 mm, 2 g mass, as controls) which was partially preened under the feathers, or a similarly sized lead chest plate (Fig. 6) of 5% body mass (~ 1.2 mm thick, 18–26 g, adjusted with small additional amounts of plasticine for each bird). Three days later, this design was repeated but with thicker lead plates (~ 2.4 mm) adjusted to 10% of the bird’s body mass (17 and 18/02/2003). The results show that 11 pigeons successfully completed paired flights with working ECG recorders and analysable data for each experiment, with 8 birds completing all 4 days.

**Figure 7:**
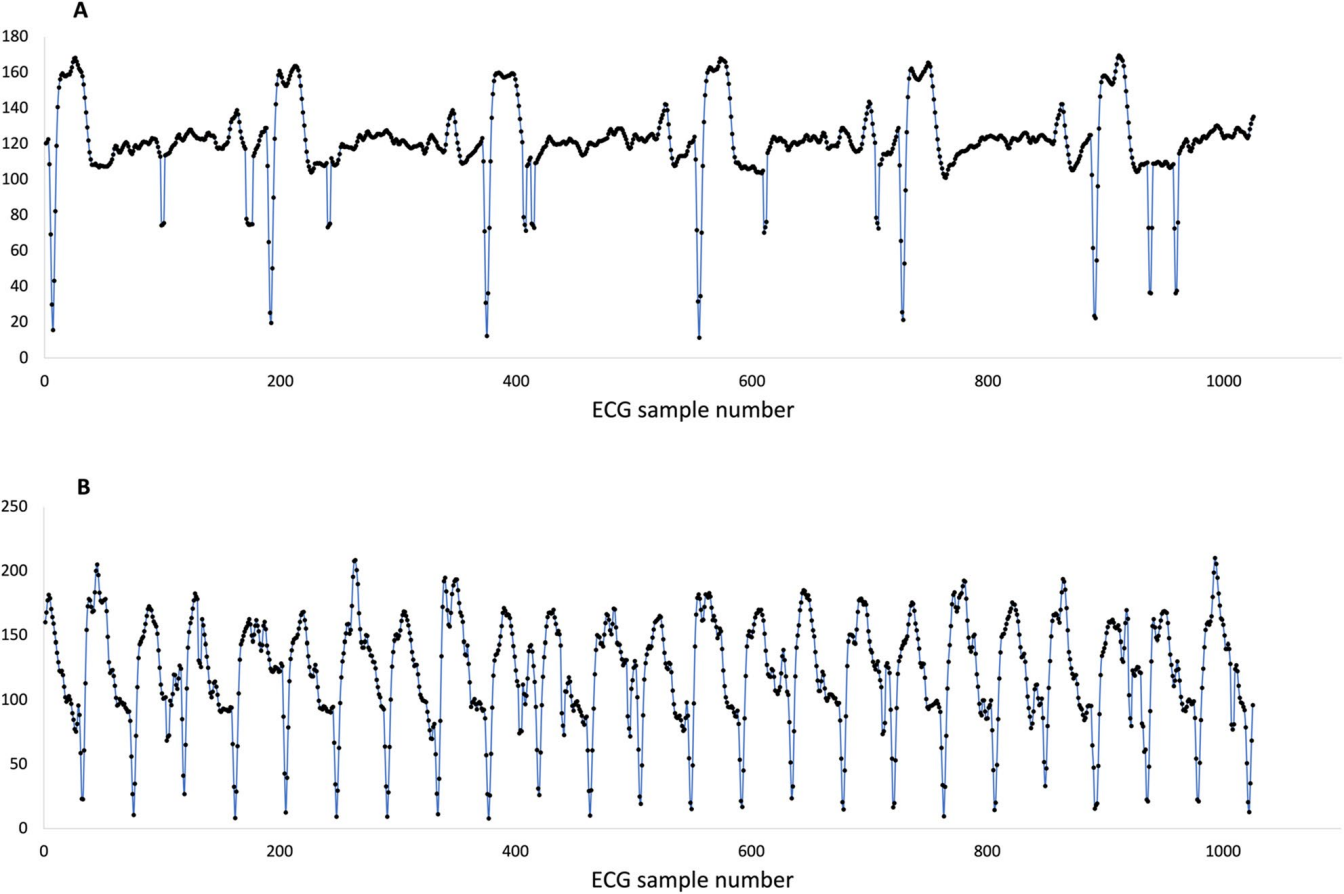
ECG sampled from Bird 93 instrumented on 18th February 2003 at 512 Hz. (**A**) ECG while waiting in the travel crate with heart rate of around 165 bpm before release and (**B**) ECG towards end of homing flight with heart rate just over 700 bpm.

**Figure 8:**
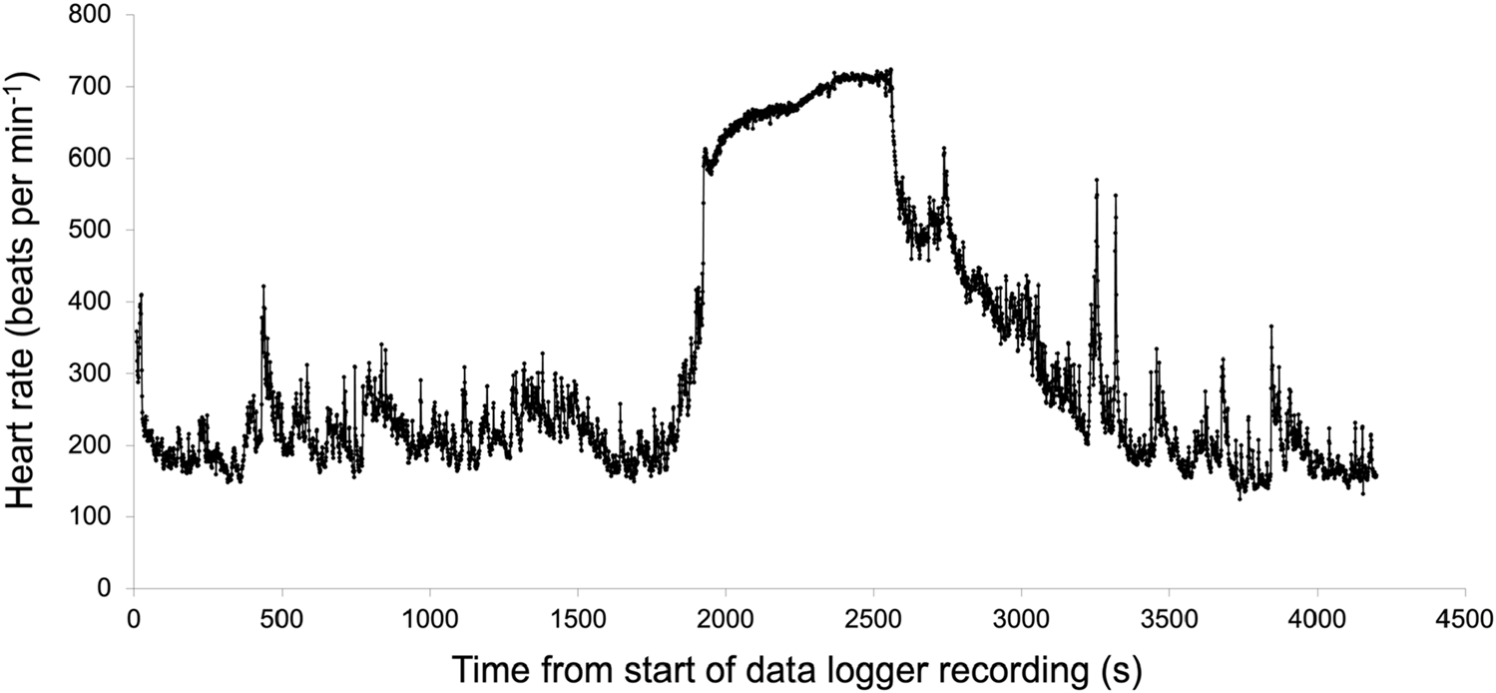
Heart rate recorded from Bird 93 flying on 18th February 2003.

**Figure 9:**
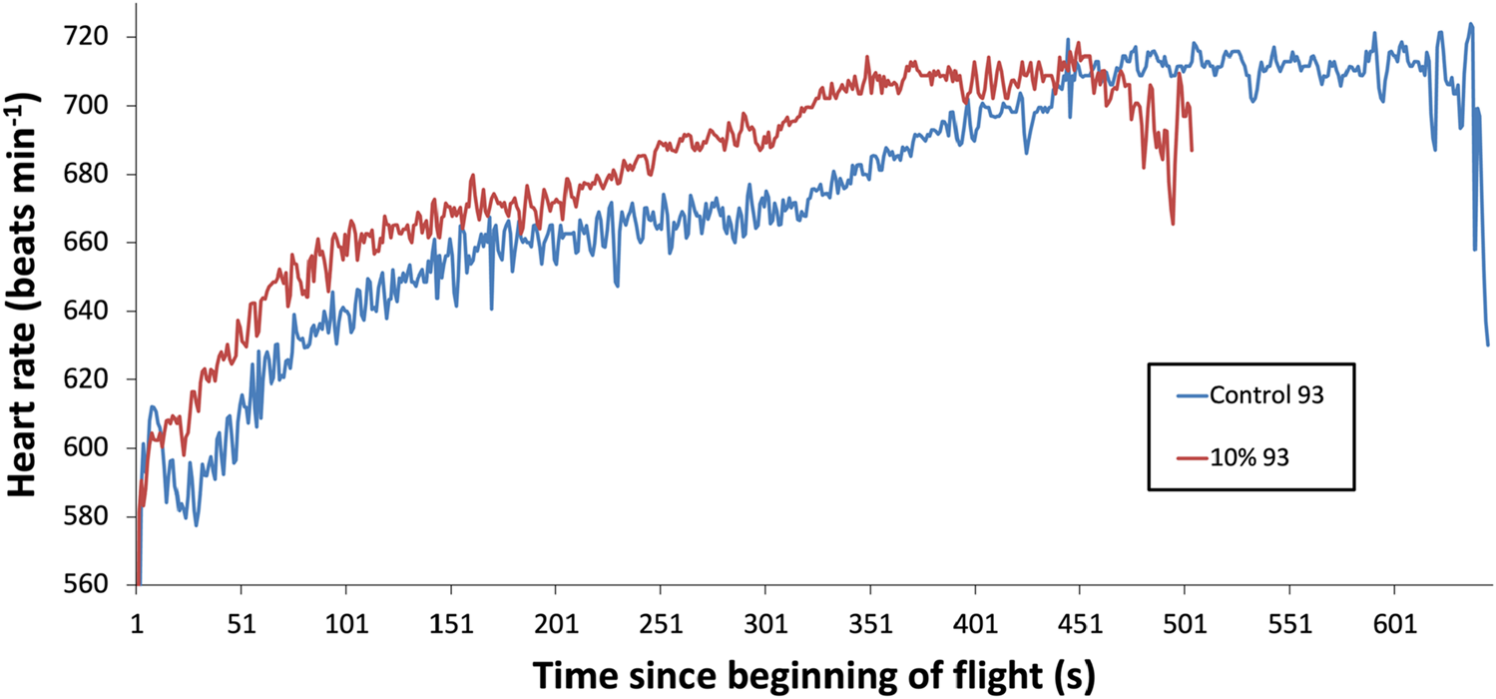
Heart rate recorded from Bird 93 flying on 17th and 18th February 2003.

ECG was sampled at 512 Hz and resulting data files were downloaded to a PC, plotted (Fig. 7A,B), gently smoothed with a rolling 3-sample average (Data S1). Data was decoded using Bangor University software, optimised to detect the QRS peak of the ECG waveform. Inter-beat R–R intervals were measured and converted into instantaneous heart rate (beats per minute, bpm) and reported every 1 s (Fig. 8—Data S2, Data S3). Heart rate data were smoothed by taking the median bpm within a rolling 5 s interval (Data S4, Data S5) and plotted as within-subject pairs (Fig. 9). Independent Student’s t-tests assuming unequal variances were used to compare mean flight times on any given data between control and loaded pigeons. Median flight times between the treatment groups on a given day were relatively small, especially for the 10% loading experiment (5% load verses control—day 1 + 2.2% (t = 3.18, P = 0.138), day 2 + 6.9% (t = 3.18, P = 0.003); 10% load verses control—day 1 + 0.2% (t = 2.45, P = 0.349), day 2 − 0.23% (t = 2.45, P = 0.338)), as the pigeons generally stayed as a loose flock over these relatively short flights, thus variation in flight velocity was not a serious confound. The median straight-line ground speeds from the release site on the four experimental days were calculated as 22.1, 18.3, 19.96 and 16.1 m s^−1^, respectively. For modelling purposes, an airspeed of 21 m s^−1^ was used. This was determined from the average of many subsequent flights for pigeons instrumented with GPS tags and flying in similar flocks back to the Treborth Loft^14^, and calculated using measurements of local wind strength and direction, taken from the Welsh Traffic Control anemometers located on the Britannia Bridge, near the loft.

Mean heart rate measurements are converted to estimates of mean rate of oxygen consumption, adjusting for flight body mass and assuming a heart mass of 1.29% of body mass^8^. Estimated rates of oxygen consumption were plotted against flight mass for each pigeon (when loaded and when acting as a paired control) and the mass-exponent calculated, to yield a mean ± SEM exponent for 5% and 10% loaded birds of 0.809 ± 0.210 and 0.667 ± 0.183, respectively, which were not significantly different from each other (t = 2.16, P = 0.54, n = 11).

### Ethical approval

All use and care of animals, homing flights and specific experimental protocols used in this study were conducted and approved under a UK Home Office ASPeL Project and Personal licence to CMB, following review and approval of the Animal Ethics and Welfare Committee of Bangor University, UK. This is compatible and equivalent to the ARRIVE guidelines.

### Supplementary Information

Supplementary Information 1.

Supplementary Information 2.

Supplementary Information 3.

Supplementary Information 4.

Supplementary Information 5.
